# Methodology to Validate the Radiated Immunity of Sophisticated Automotive Autonomous Systems †

**DOI:** 10.3390/s25041244

**Published:** 2025-02-18

**Authors:** Nadir Fouad Bedjiah, Moncef Kadi, Marco Klingler, Romain Rossi

**Affiliations:** 1 Stellantis, Centre Technique de Vélizy Route de Gisy, 78943 Vélizy-Villacoublay, France; marco.klingler@outlook.com; 2 ESIGELEC, Institut de Recherche en Systèmes Electroniques Embarqués (IRSEEM), Technopôle du Madrillet, 76000 Rouen, France; moncef.kadi@esigelec.fr (M.K.); romain.rossi@esigelec.fr (R.R.)

**Keywords:** EMC, radiated immunity, complex systems, automotive systems, autonomous vehicle, autonomous systems, semi-anechoic chamber, component radiated validation, ADAS

## Abstract

The trend in all automotive manufacturers is to commercialize vehicles with an increasing number of sophisticated Advanced Driver-Assistance Systems (ADASs). These systems often require that several sensors, such as Light Detection and Ranging (LIDAR), radio detection and ranging (radar), cameras, etc., work in cooperation, which makes the systems very complex. To perform the electromagnetic compatibility (EMC) validation of these complex ADASs, the stimulation of multiple sensors composing the system is necessary. Furthermore, the synchronization of these stimulations is essential to create realistic outdoor scenarios in the usual EMC facilities (on a roller bench in a semi-anechoic chamber). This synchronization is mandatory as the integrated safety systems will disable any ADAS or autonomous system in case of incoherencies in the data delivered by the sensors, rendering the validation challenging. Moreover, the current methodologies proposed are meant to be performed to validate simple ADASs based on simple sensors. In addition, with the current test facilities, one cannot stimulate, in a realistic and synchronous way, multiple sophisticated sensors (e.g., LIDARs and inertial measurement units). For all these reasons, the radiated immunity tests of future automotive systems will be endlessly difficult following current trends. In addition, the complexity of the systems and their increasing number increase the duration and cost of these immunity tests and make their validations more challenging. In this article, we present a new methodology to validate the radiated immunity of complex automotive autonomous systems to address these challenges. The results we present show that this new methodology can be performed to validate ADASs and autonomous automotive systems independently of their complexity.

## 1. Introduction

The complexity of electrical/electronic (EE) architectures in the automotive domain is constantly growing with the complexity of autonomous systems and future Advanced Driver-Assistance Systems (ADASs) [1,2,3,4,5,6,7]. Currently, validating and approving a vehicle’s radiated immunity requires compliance with international and/or continental standards, such as ISO 11451 and ECE R10 [8,9,10,11,12]. There are two types of radiated immunity tests: bench (on-table) tests, which validate individual electronic components such as sensors, actuators, and ECUs in compliance with ISO 11452 standards [13,14], and on-vehicle tests, which assess the performance of an integrated system comprising multiple electronic components [15,16,17,18]. Thus, the on-vehicle tests validate automotive systems such as ADASs and autonomous systems relying on electronic components already validated on-table, and working together as a system [19,20]. Nowadays, ADASs can be classified into two types: simple ADASs, which rely on one or two sensors, and complex ADASs, which require the synchronization of multiple sensors. The radiated immunity validation of simple ADASs is already being performed. It involves stimulating the sensor and monitoring the system’s response to these stimulations [21,22]. The monitoring can be performed essentially by camera or by the diagnostic link. To validate autonomous systems and complex ADASs based on multiple sensors, the current trend is to try to create a realistic outdoor scenario in a semi anechoic chamber by stimulating, in a realistic and synchronous way, the sophisticated sensors (e.g., LIDARs, radars etc.) in real time [23,24]. The monitoring of such complex systems is identical to the monitoring of simple ADASs. However, this validation is challenging since the synchronization of these stimulations is mandatory, which requires the development of a multiple sensor and communication (5G, V2X, GPS, etc.) stimulator metasystem. Any inconsistencies between these stimulations will result in discrepancies in the data provided by the communication links and sensors. These inconsistencies will cause the autonomous system to disable itself for functional safety reasons, making EMC validation in autonomous mode impossible. This challenge will become even more significant as self-monitoring algorithms continue to improve in intelligence. To prevent such situations in the future, we have developed a new methodology to validate the radiated immunity of advanced ADASs and autonomous systems. This methodology is based on the implementation of specific validation test software algorithms to perform a succession of simple component radiated immunity validation tests at system level to validate a complex system, as described and validated with AM modulation in [25], while remaining non-intrusive from an EMC and electromagnetic perspective. The methodology was developed to be performed on-vehicle, on a roller bench in a semi-anechoic chamber. This methodology allows the validation of complex systems in fewer steps and avoids the development of a highly costly metasystem to recreate outdoor scenarios in a semi-anechoic chamber.

In this paper, we will present the new methodology’s principle and present the test results carried out both with the traditional methodology and our proposed methodology on-table in continuous wave (CW) mode. In addition, as our methodology is developed and meant to be applied on-vehicle, we present the validation of this methodology’s principle with on-vehicle tests also in CW mode.

This paper is organized as follow: In Section 2, we present the current trend to validate ADASs and future autonomous systems. In Section 3, we present our new methodology principle. In Section 4, we describe the demonstration EE architecture developed, and the test bench made to develop the new methodology. Finally, in Section 5, we present and analyze the obtained results.

## 2. Immunity Validation of ADASs, Complex, and Autonomous Systems (Traditional Methodology)

**Figure 1:**
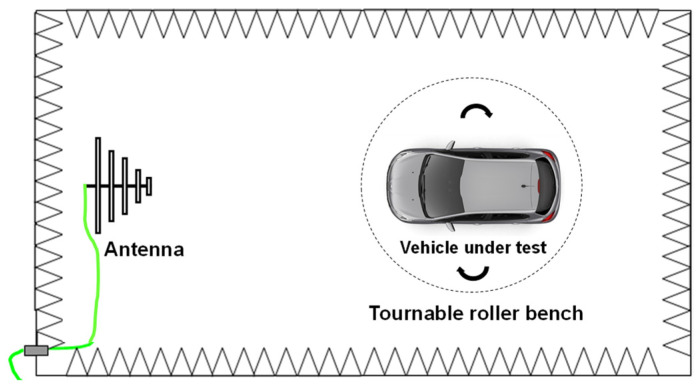
Setup of radiated immunity tests [26].

In this section, we highlight the principle of the traditional method to validate ADAS systems. In this method, the radiated immunity validation tests of automotive systems are performed following standard ISO 11451 [13], on a roller bench in a semi-anechoic chamber (Figure 1). An antenna is placed as source in front of the vehicle under test in vertical or horizontal polarization. The radiated immunity tests are carried out over a given frequency band, increasing, at each frequency step, the field level in search of the threshold field level of the system under test, by monitoring errors at actuator levels and/or at the vehicle dashboard level (displayed indicators). If a malfunction is detected at a given frequency, one proceeds to a fine search of the minimum field level and leads an investigation on-vehicle to determine which component is faulty before passing to the next frequency. The current trend to validate these systems is to stimulate several sensors and monitor how the systems respond to these stimulations by monitoring the actuators’ responses and/or the indicators displayed on the dashboard.

**Figure 2:**
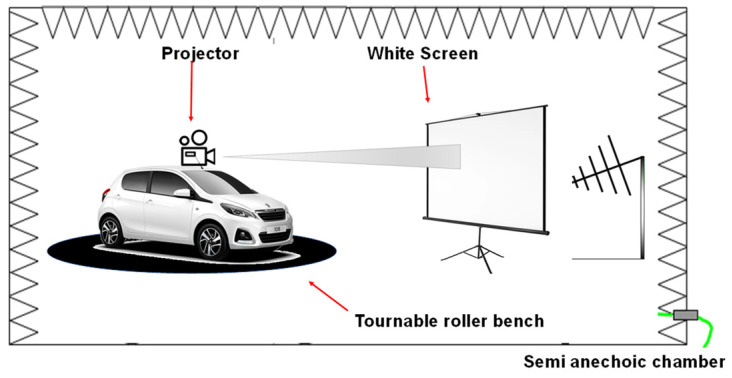
Example of a radiated immunity validation test of a traffic sign recognition ADAS.

In Figure 2, the radiated immunity validation test of a traffic sign recognition ADAS based on the use of the front camera is presented as an example of the traditional methodology of validation. The vehicle under test is placed on a roller bench in a semi-anechoic chamber. To validate this system, one stimulates the camera with a video projector that displays a succession of speed-limit traffic signs on a white screen placed in front of the vehicle and monitors the speed-limit signs shown on the vehicle’s dashboard. If the ADAS relies on multiple sensors, we applied the same principle to validate the system and added synchronization to the stimulation of these sensors, which can be a limitation given that there is no existing metasystem that allows synchronization of all stimulations.

Furthermore, to validate the radiated immunity of the EE architecture and the complete system, we currently validate the immunity of each individual system. However, these systems rely on several common sensors and actuators, as outlined in Table 1. The existing procedure involves testing each system separately, and for more sophisticated systems, synchronous stimulation is required.

In Table 1, we present a simplified example of six ADASs or autonomous systems sharing six ECUs embedded in a vehicle, which together comprise the complete EE system. Each system must be validated as described for the traffic sign recognition system, with the added requirement of synchronizing stimulations. To validate the immunity of the vehicle’s EE architecture, based on ECUs 1 to 6 and embedded functions 1 to 6, we validate each system individually (Systems 1 to 6).

For instance, ECU 4 will be tested during the validation of System 1, System 2, System 4, and System 6. If malfunctions are detected while validating System 4, we must investigate to identify which component is not meeting the immunity requirements. If, for example, two components (ECU 1 and ECU 5) are faulty within the same frequency band but at different field levels, the investigation will lead only to the component with the lowest susceptibility field level (ECU 1 in this case). This faulty component will then be sent to the supplier for improvement.

Once ECU 1 is returned, we will repeat the validation immunity tests and detect any other malfunctions in the same frequency band at a higher immunity level corresponding to the susceptibility of ECU 5. The investigation will then continue, following the same procedure, to detect and address the faulty component.

The traditional methodology requires a metasystem that realistically stimulates all embedded sensors to validate complex ADASs or autonomous systems. Additionally, multiple validation steps are needed to identify faulty sensors if they malfunction at the same frequency but not at the same field level. Thus, the new OVCV method eliminates these application challenges and issues. The following section presents the proposed methodology.

## 3. On-Vehicle Component Validation Methodology for Immunity of Complex Automotive Systems

### 3.1. Methodology Principle

Instead of validating the complex systems one after another, we propose to use, during the validation tests, specific test software programs implemented in each component, in order to validate all the components one by one at system level directly on-vehicle, and therefore to validate globally all the systems at the same time. These test software programs will be specifically developed to be able to validate each ECU separately depending on its tasks.

Our new methodology is based on two hypotheses from a hardware and software point of view. First, from a hardware point of view, the EM and electrical behavior will be the same if the hardware is not modified. The test shall be physically non-intrusive (supply, grounding, position, and orientation of the components, equipotential). In addition, the central ECUs shall be functional if they interface with other ECUs. Second, from a software point of view, if the software does not contain any digital filtering, a test software shall be developed to activate all the electronic chips in the component in such a way as to cover any other software algorithm. If the software contains digital filtering that improves the immunity performances of a component, test software without filtering shall cover the worst case in immunity.

**Figure 3:**
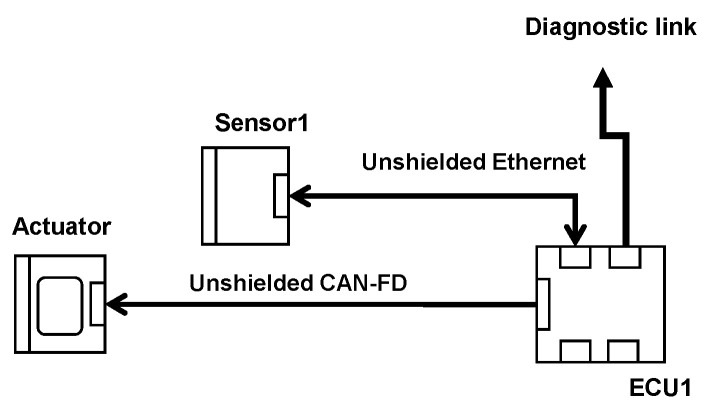
Example of EE architecture.

In Figure 3, an EE architecture is described. It is based on a sensor, an electronic command unit (ECU), and an actuator. To validate this architecture with the traditional methodology, we stimulate Sensor1; depending on these stimulations, the sensor generates its own data that are sent to ECU1, which, in turn, generates data (or commands) that are sent to the actuator. The actuator generates its data depending on the data sent by the ECU. The monitoring of the system is made at actuator level, by monitoring the responses or data provided by the actuator. The tests follow the procedure described above in Section 2. If we detect malfunctions during the test, we should proceed to an investigation to find the faulty component(s). Once all faulty components are found, we improve them and perform the same test procedure to ensure that no more errors are detected.

**Figure 4:**
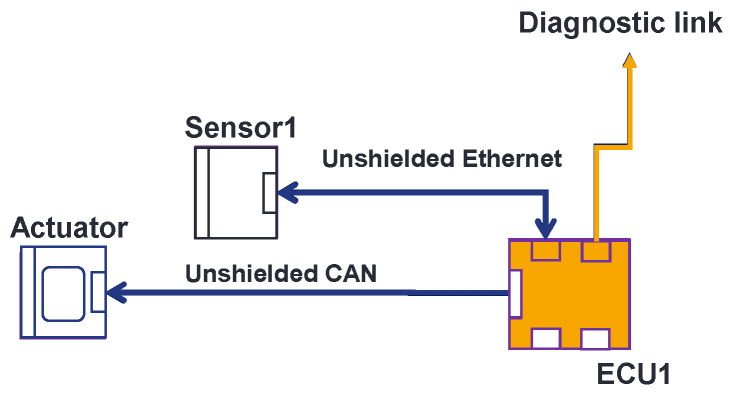
Component validation of ECUs.

**Figure 5:**
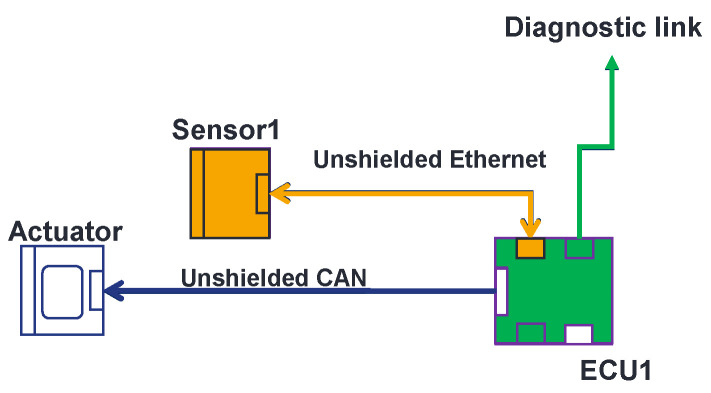
Component validation of sensors.

**Figure 6:**
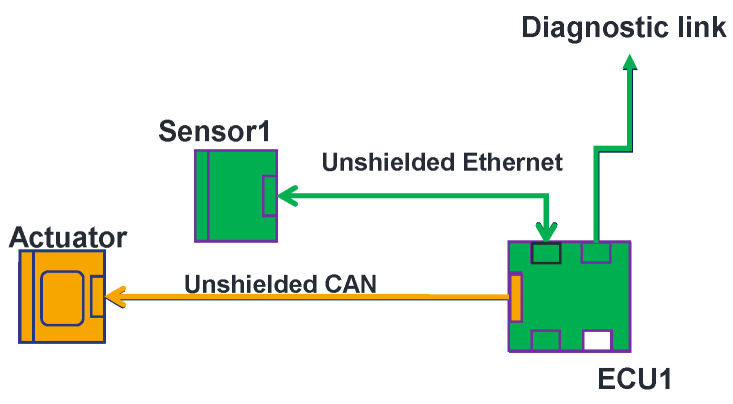
Component validation of the actuator.

The OVCV’s principle is simple. By implementing test software in each component, one performs component validation of several electronic components at their real position in the vehicle with their real communication links at system level. Take, for example, the EE architecture described in Figure 3. The validation of the ECU is the first step to perform OVCV tests. We use a test software that is implemented in the ECU that allows the ECU to send known data to the diagnostic link (see Figure 4) on which the monitoring is performed. If the ECU does not comply with the immunity requirements, all the systems relying on this ECU cannot be not validated and the susceptibility levels of the ECU will be communicated to the supplier to improve the component. This is already the case with the conventional methodology. Once the ECU is returned by the supplier (in the same validation phase or the next one), and it then complies with the requirements, we switch to a second software test program implemented in the ECU that allows us to send received data from the sensors directly to the diagnostic link. With this second software program, we can therefore validate the sensor. We stimulate the sensor and, knowing the data that should be generated, we can monitor the integrity of data generated and response delay at the ECU’s diagnostic link level (see Figure 5). Finally, to validate the actuator, we switch to a third test software program implemented in the ECU. This software allows the ECU to send known commands to the actuator. Knowing the command sent by the ECU, we can monitor the good functioning of the actuator, its responses, and its response delay (see Figure 6). Following this methodology, we validate all the communication links of the components and all their central processing units (CPUs). With the ECU’s component validation, the CPU of the ECU and diagnostic link are validated. With the sensor’s component validation, the physical sensor, the CPU, and the communication link of the sensor are validated. The communication link of the ECU that is linked to the sensor is validated at this step. Finally, with the component validation of the actuator, one validates the CPU, communication link, and the optical link of the actuator. We also validate at this step the communication link that connects the ECU with the actuator. During the validation of the ECU, the sensor, the actuator, and, eventually, others, components continue to be active, as is the case in a real architecture, but the monitoring during the immunity tests does not take into account the state of the components that we are not validating. Thus, with the OVCV methodology to validate the EE architecture described in Table 1, we validate ECU1 to 6, applying the OVCV methodology principle to validate the complete complex EE architecture embedded and thus all the systems (System 1 to 6).

### 3.2. Procedure to Validate the New Methodology

**Figure 7:**
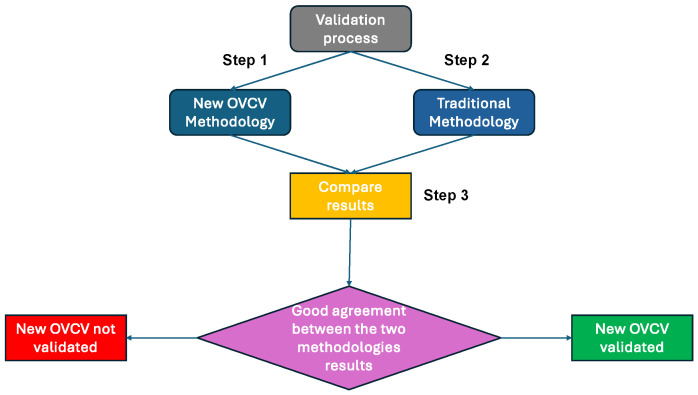
Procedure to validate the new methodology principle.

The procedure to validate and develop the new methodology is described in Figure 7. A first, radiated immunity validation of a first version of the complete EE architecture with the traditional methodology is performed. The susceptibility curve obtained at this step is used as a reference. The second step is to perform the tests with the On-Vehicle Component Validation methodology principle. We start this step by performing component validation at system level for all the components that are used to interface with other components, such as the ECUs (see Figure 4) at their real position in the architecture in the vehicle with their real communication links. After that, we perform component validation of the sensors and actuators (see Figure 5 and Figure 6). The minimum of the susceptibility curves obtained with the OVCV methodology is compared with the susceptibility curve obtained in step 1 performing the traditional methodology. If we obtain identical results and have good agreement between the two methodologies’ results, considering measurement uncertainties, the new methodology is validated. If we do not obtain a good agreement between the two methodologies’ results, the methodology is not validated. The only modification of the EE architecture between the two methodologies is the software implemented in the generic components. We use specific test software programs in each component to perform the OVCV methodology and the final intended software (which would correspond to the software implemented when the vehicle is marketed) to perform the traditional methodology as reference.

## 4. Representative Electronic/Electric (EE) Architecture Development

In this section, we describe the setting up of a test bench to develop the new methodology to validate the complex autonomous systems of future vehicles. The test bench is composed of an EE embedded architecture part and a stimulation and monitoring part.

### 4.1. Electrical and Electronic (EE) Architecture Development

Autonomous vehicles are currently in the prototype stage. Using a real EE architecture to validate the new methodology is challenging. In addition, the real components used in the real architectures do not allow us to modify the software in the architecture to use test software programs in a simple way. These components are used as black boxes by car manufacturers. Thus, an electrical and electronic architecture was developed to validate the new methodology. The EE architecture was developed in a way to have the capability to develop our own software (test software and final software programs), and to adjust and configure each specific test software, which allows for the development, test, and validation of the new methodology.

**Figure 8:**
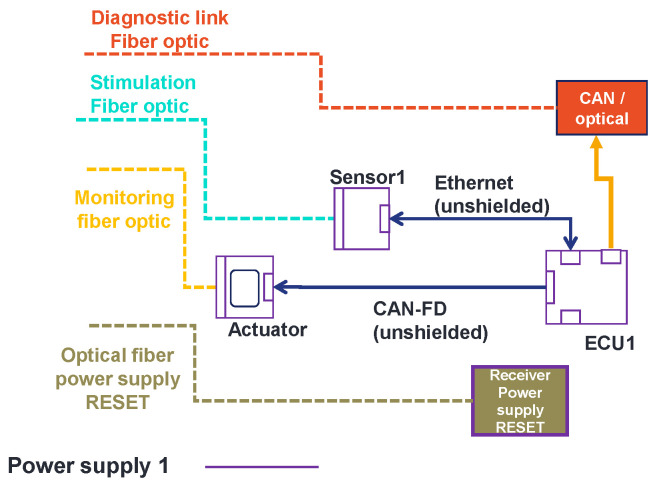
Architecture under test.

**Figure 9:**
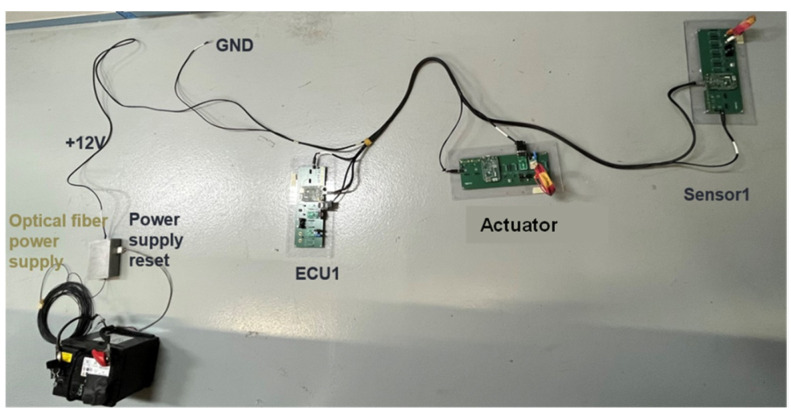
Real architecture under test.

In Figure 8, we present the architecture under test. It is made of generic electronic components that we developed, the receiver of the power supply reset (which allows us to remotely reset the architecture), and a CAN-to-optical converter. The architecture is composed of a sensor, an actuator, and an ECU (Sensor1, ECU1, and actuator). To connect the components together, an automotive cable harness was made (see Figure 9). Sensor1 is connected to ECU1 with an ethernet link. The ECU is linked to the actuator with a SUB-D9 link. The aim of using this EE architecture is to validate the principle of the methodology.

As it is described in Figure 9, all the components are supplied with a 12 V automotive battery through a power supply reset system with 12 V jack connectors through the same harness. As is the case in a real ECU, the generic ECU has a diagnostic link to monitor eventual errors. A ground port is also available to connect the architecture to the ground plane.

### 4.2. Electronic Architecture of Generic Components

**Figure 10:**
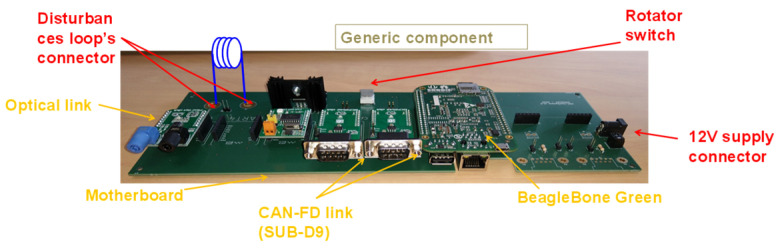
The component’s hardware architecture.

In this part, the electronic architecture of generic components is described. All the components have the same electronic architecture (Figure 10). We chose this approach to master the hardware and the software development of the architecture of the prototype. The generic components are composed of a single-board computer with three communication connectors (an ethernet connector, USB A connector, and micro-USB connector). The components are also composed of communication boards, for communication links that are not available on the single-board computer, such as CAN link, fiber optical link, etc. For demonstration purposes only, we used optical fibers to monitor a set of generic components when they are configured to act as actuators, and to stimulate another set of generic components when they are configured to act as sensors. This allows us to avoid having to develop sets of different sensors and actuators based on different technologies, which would not have had any added value with respect to the purpose of this demonstration. Of course, once this methodology has been validated and applied to a real vehicle, the embedded sensors and actuators will be, respectively, stimulated and monitored with appropriate interfaces, as is already carried out in conventional testing.

To connect the single-board computer to the communication board, we developed a motherboard. The motherboard is composed of a 12 V jack to power the components and a rotatory switch for switching between the different roles (from ECU role to sensor role, from sensor role to actuator role, etc.).

In this motherboard, we dedicated a specific connector to connect the disturbance loop to the generic components in such a way as to create malfunctions for each component in different frequency bands and at different levels of susceptibility. The motherboard, the communication link, the Beaglebone board, and the disturbance loop are considered generic components.

### 4.3. Optimization of Disturbance Loops (Disturbance Loop Modeling)

To be able to finely control the susceptibility of the components of our test system, disturbance loops have been designed and introduced in each component. The connectors for the component disturbance loops are shown in Figure 10. The proposed loops were made to give a different susceptibility signature to each component in terms of frequency bands and level of susceptibility. To develop the disturbance loops, we calculated the diameter and the number of turns of the loops depending on the frequency band and the field level in which we wanted the loops to create faults in the generic component.

**Figure 11:**
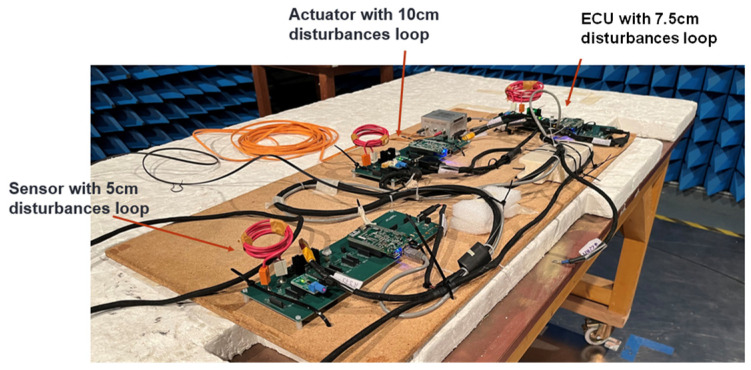
Electronic components with disturbance loops.

To disturb several generic electronic components, we used disturbance loops of different diameters and different numbers of turns. The ECU’s loop is 7.5 cm in diameter with eight turns. The actuator’s loop is 10 cm in diameter with 10 turns. Finally, the loop used for the sensor is 5 cm in diameter with six turns (see Figure 11).

**Figure 12:**
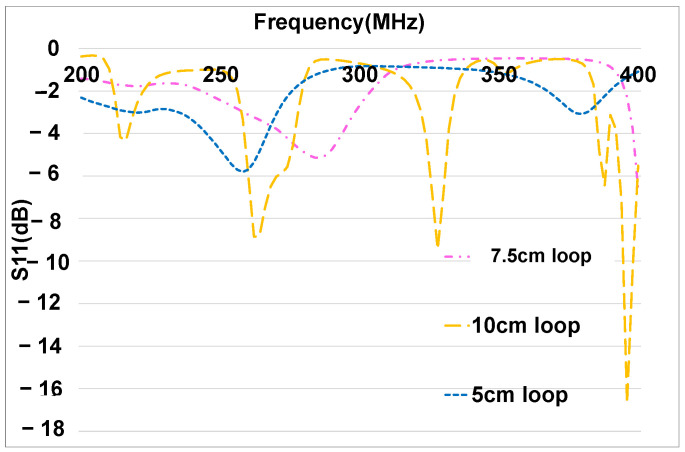
Measurement of disturbance loops’ reflection coefficient (S11).

**Figure A1:**
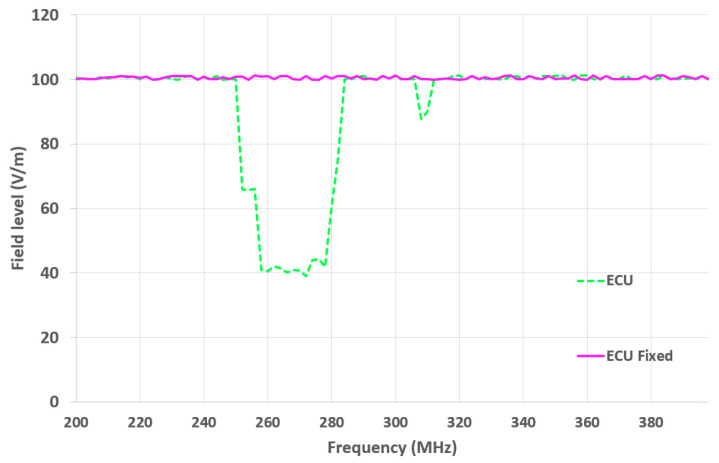
Susceptibility curves of the ECU with and without disturbance loops.

In Figure 12, the measured S11 parameter of each loop is presented. Several loops and equivalent antennas resonate at different frequency bands with different levels of resonance. Each loop allows us to disturb the components in different frequency bands during our study. The ECU’s loop has multiple resonances at 210 MHz, 260 MHz, 310 MHz, and around 385 MHz. The actuator’s loop has resonances at 200 MHz, 250 MHz, and 390 MHz. Finally, the sensor’s loop has resonances at 240 MHz and around 340 MHz. As described in Figure A1, the components developed are not faulty without the disturbance loops. Thus, the faults that occur during testing are due to the disturbance loops. 

### 4.4. Stimulation and Monitoring of the EE Architecture

**Figure 13:**
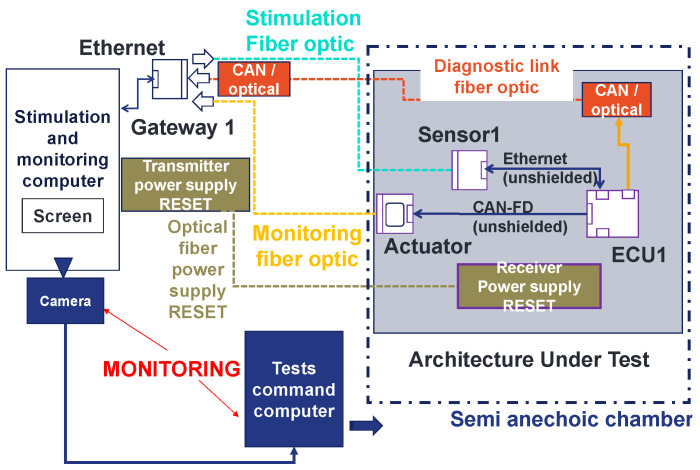
Test bench to develop a new methodology to validate complex and autonomous automotive systems.

**Figure 14:**
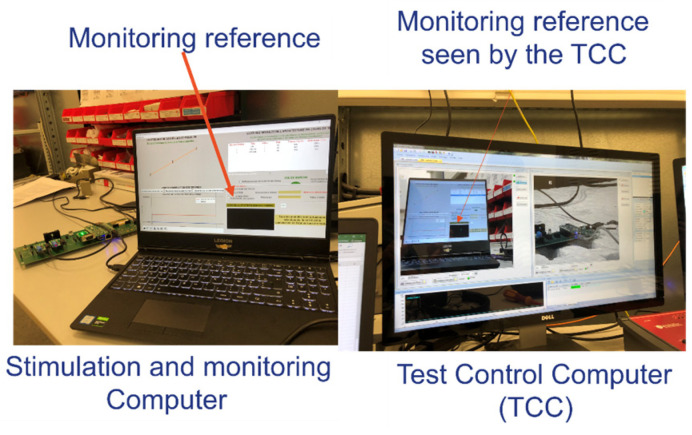
The stimulation and monitoring parts of the test bench.

The designed test bench is divided into two parts: the architecture under test (or embedded EE architecture) and the stimulation and monitoring part. The embedded architecture was presented in Figure 8. In this part, the stimulation and monitoring part is described. First, this part is composed of a stimulation and monitoring computer (SMC). This computer is used to stimulate the sensors and monitor the data provided from the architecture, specifically, the actuators. In addition, this stimulation and monitoring part has a gateway which is a specific mode of the generic components. Its role is to convert data sent by the SMC to the architecture from the ethernet to the Universal Asynchronous Receiver Transmitter (UART) protocol using optical fiber. The gateway also converts the UART and CAN data provided from the architecture to ethernet data. In this way, the SMC can stimulate and monitor the architecture. In addition, the stimulation and monitoring part has a power supply reset transmitter, which allows for the resetting and rebooting of the architecture by pushing a push button. This allows us to avoid entering the semi-anechoic chamber each time an error occurs during the tests that require to perform a hardware restart of the architecture. Finally, an EMC test control computer connected to a camera is described in Figure 13. There is a camera connected to the test command computer oriented to the SMC screen. In cases where the SMS detects malfunctions in the embedded architecture, a part of its Human-Machine Interface (HMI) turns from black (no error) to white (error detected) and the test command computer detects the change through the camera and suspends the radiation (see Figure 14). At this step, the reset button is pushed and the field level threshold is investigated.

**Figure 15:**
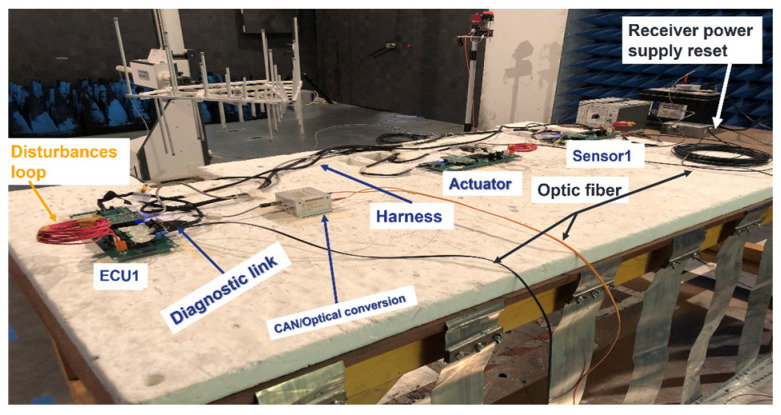
Setup for the on-table tests (bench tests).

In Figure 13, four optical fibers are presented. The first fiber is dedicated to the stimulation of the sensor. A second fiber is used for monitoring of the actuator level. There is a third fiber for the power supply reset. Finally, there is an optical fiber for the diagnostic link connected to the CAN-to-optic converter. The actual position of the optical fibers is shown in Figure 15.

## 5. Analysis of Immunity Test Results

In this section, the results of the radiated immunity tests are presented. To validate the methodology’s principle, we followed the validation process described in Figure 7. We started with the on-table validation tests for their simplicity with respect to validating the principle of the new methodology. Once the principle of the new methodology was validated on-table, we performed on-vehicle validation, given the methodology was developed to be only performed for on-vehicle validations.

### 5.1. On-Table Tests (Bench Tests)

In this part, the on-table setup and tests results are described. The first test results for an AM modulation were presented in our previous work for the 2022 IEEE symposium [9]. The results presented below were obtained for a CW.

In Figure 15, the setup of the on-table tests is described. The architecture under test is composed of an ECU, a sensor, and an actuator. We have four optical fibers: one for the stimulation of the sensor, one for the diagnostic link, one for the monitoring of the actuator, and one for the power supply reset. The antenna was placed in front of the table 1000 mm from the actuator. The architecture was placed at a height of 50 mm from the ground plane. We performed the test in the frequency band 200–400 MHz with a target field level of 100 V/m. CW without modulation was applied in these tests. The ground connector of the EE architecture was connected to the ground plane of the table. The results presented in this paper were obtained for vertical polarization. The threshold field level research is performed using a 1 dB field step.

**Figure 16:**
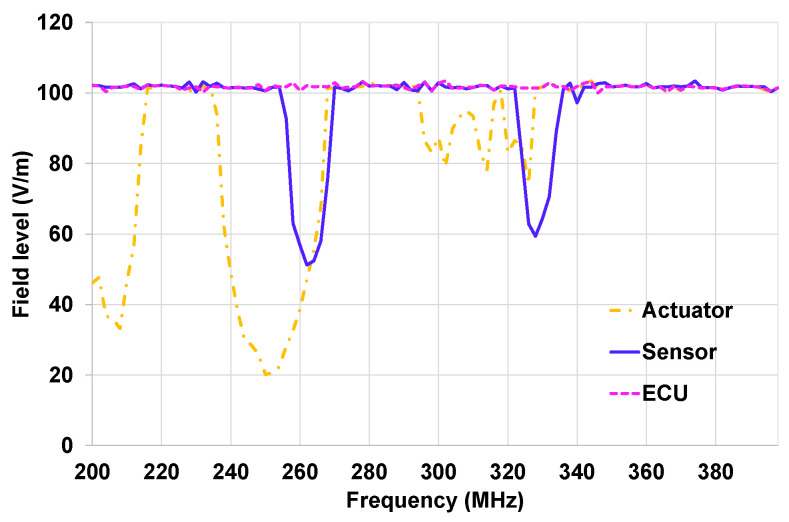
Susceptibility curve of On-Vehicle Component Validation tests for on-table tests.

In Figure 16, the susceptibility curves of each component validation at system level with the OVCV methodology are presented. Disturbance loops were connected to the actuator and the sensors. The ECU was free of a disturbance loop. The sensor and the actuator of the EE architecture were validated using the ECU as an interface following the OVCV’s principle. A specific test software was implemented in the EE architecture’s components.

We can see through the results described in Figure 16 that the ECU shows no malfunctions in the frequency band 200–400 MHz, and the actuator has several frequency bands where malfunctions were detected: 200–214 MHz, 236–266 MHz, and 294–326 MHz. Malfunctions were detected during the sensor’s validation in the frequency bands 254–268 MHz and 336–346 MHz.

**Figure 17:**
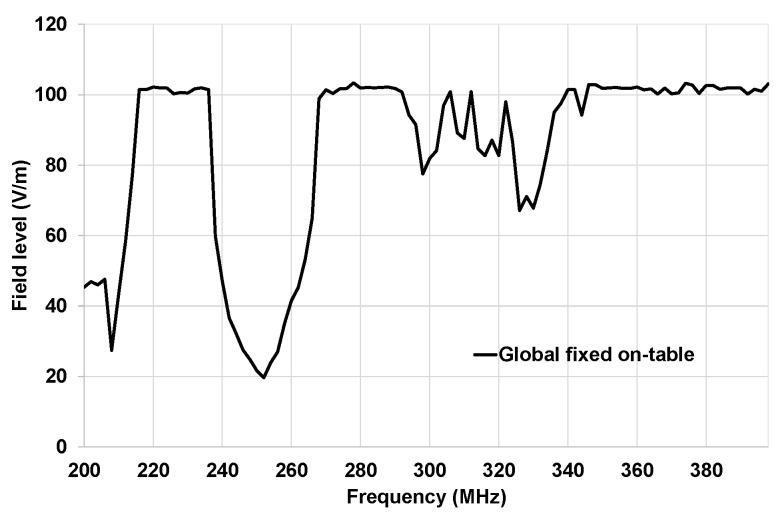
Susceptibility curve of the complete architecture for on-table tests.

In Figure 17, the susceptibility curve of the complete architecture is described. We applied the traditional methodology to validate the radiated immunity of the complete EE architecture. We used the same architecture that we used for the OVCV tests. The disturbance loops were connected to the sensor and the actuator. The disturbance loop was removed from the ECU. As explained in Section 3, the software in this case is the final intended software, including the programs performing the following tasks: The sensors receive stimulation data from the monitoring system through its optical interface. It then generates its own data depending on the data received and sends its data to the ECU. In turn, the ECU generates its own data depending on what it has received from the sensor and sends its own data and the data received to the actuator. Finally, the actuator generates its own data depending on what it receives from the ECU and communicates its data to its optical interface. These data are then checked by the monitoring system, which expects the correct data according to what it has sent to the sensor. We can observe that malfunctions have been detected in several frequency bands: 200–212 MHz, 236–268 MHz, and 294–346 MHz.

The susceptibility curve described in Figure 17 is considered as a reference curve and will be compared to the minimum susceptibility obtained with the succession of component validations at the system level (described in Figure 16) following the OVCV methodology’s principle.

**Figure 18:**
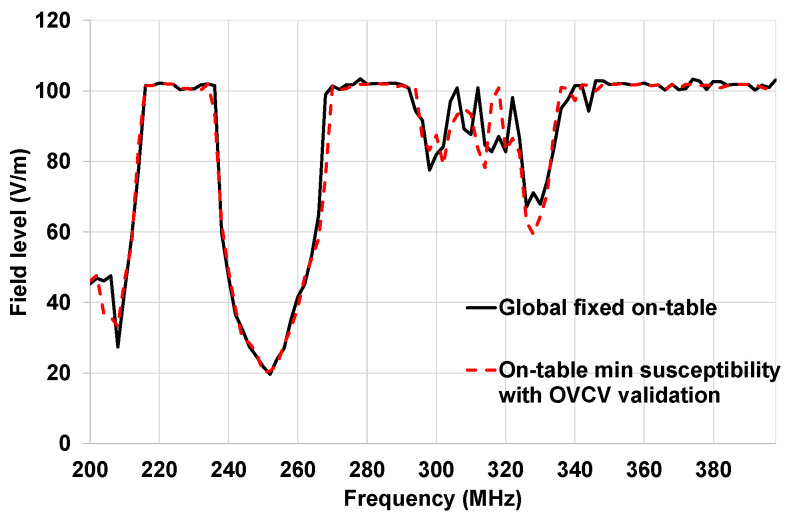
Comparison of the susceptibility curves obtained with the traditional methodology (ECU improved) and the minimum susceptibility curve of OVCV for on-table tests.

In Figure 18, the susceptibility curve of the complete architecture, obtained with the traditional methodology, is compared to the minimum susceptibility obtained with the OVCV methodology (ECU, actuator, and sensor component validations combined).

The results presented in Figure 18 demonstrate that we obtained good agreement between the results obtained with the traditional methodology and the results obtained with the OVCV methodology, considering the measurement uncertainties. At this point, we can validate the principle of the OVCV methodology.

### 5.2. On-Vehicle Test Results

In this part, given this methodology was developed for on-vehicle tests, we will present the setup of the on-vehicle tests and the results of the radiated immunity validation obtained with the traditional methodology and OVCV. The same EE architecture was used for the on-vehicle tests and the on-table tests. The frequency band, target field level, research step of the threshold field level, and wave modulation were kept the same.

**Figure 19:**
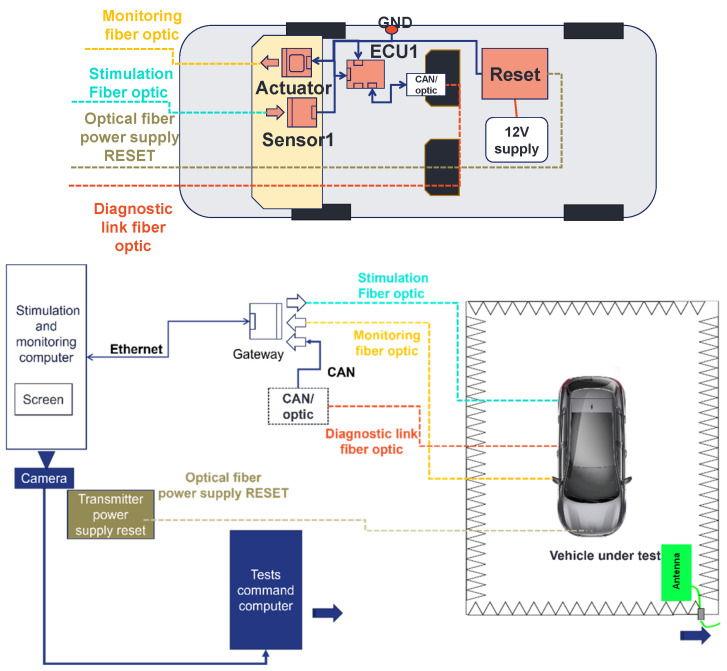
Setup for on-vehicle tests of a simple system.

**Figure 20:**
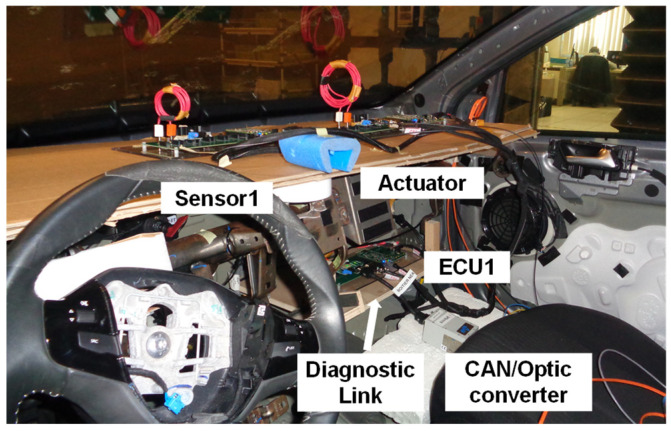
Real EE architecture used for on-vehicle tests.

Several electronic components were placed in the front part of the passenger compartment (see Figure 19 and Figure 20). The actuator and the sensor were placed at dashboard level. The ECU was placed at the glove compartment level. The same disturbance loops were kept for the actuator and the sensor as for the on-table tests. The ECU was without a disturbance loop. The receiver power supply reset and the 12 V automotive battery were placed in the rear part of the passenger compartment. The ground of the architecture was connected to the vehicle chassis.

To stimulate the architecture and to monitor it, we used the same SMC used for the on-table tests. The command of the EMC tests was performed in the same way described for on-table tests (see Figure 19).

Figure 20 describes the real EE developed architecture embedded in the prototype vehicle. We can see the actuator, the ECU, and the sensor. In addition, the diagnostic link is presented in Figure 20.

**Figure 21:**
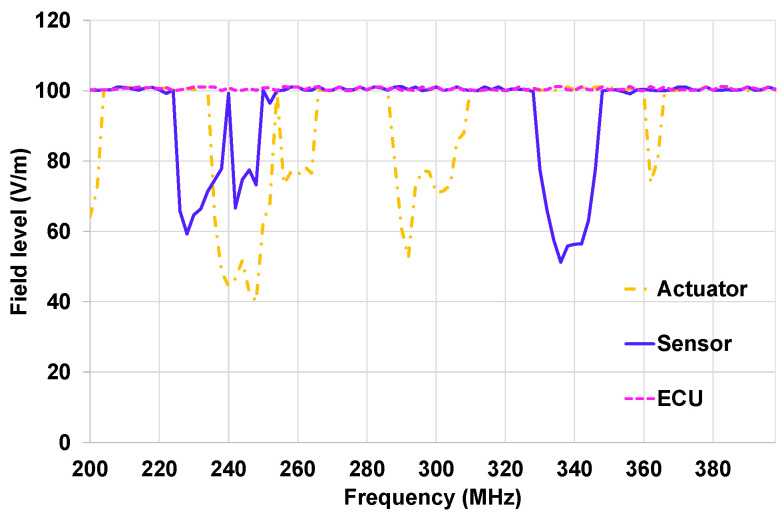
Susceptibility curve of the On-Vehicle Component Validation tests for on-vehicle tests.

Figure 21 shows the susceptibility curves of several electronic components. We performed the OVCV methodology on-vehicle to validate the ECU, the actuator, and the sensor. The ECU has no malfunctions in the frequency band 200–400 MHz. Malfunctions were detected for the actuator in the frequency bands 200–204 MHz, 236–252 MHz, 288–306 MHz, and 360–364 MHz. The sensor is faulty in the frequency bands 222–252 MHz and 328–356 MHz.

**Figure 22:**
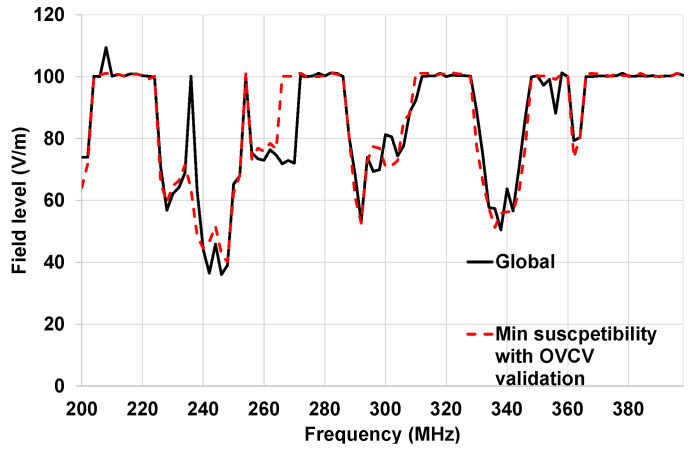
Comparison of the susceptibility curves obtained with the traditional methodology (ECU improved) and the minimum susceptibility curve of OVCV for on-vehicle tests.

In Figure 22, the susceptibility curve illustrated in red is the minimum susceptibility obtained with component validation of the generic components with the OVCV methodology.

The susceptibility curve of the complete architecture obtained with the traditional methodology is illustrated in black in Figure 22. The sensor was stimulated, and the monitoring was performed at actuator level for these validation tests. Figure 22 shows several frequency bands where malfunctions were detected (200–204 MHz, 224–270 MHz, 288–308 MHz, and 332–364 MHz).

In Figure 22, the susceptibility curve of the complete architecture obtained with the traditional methodology is compared with the minimum susceptibility obtained with the new OVCV methodology. We obtained a good agreement between the results obtained with the OVCV methodology and the results obtained with the traditional methodology, considering measurement uncertainties. The ECU was free of disturbance loops when performing the two methodologies.

There are some differences between the two methodologies in the frequency band 260–266 MHz. At the time we performed the on-vehicle tests, automation with respect to the detection of errors was not available. Thus, a test operator had to detect the fault displayed by the SMC, to suspend the tests, and to save the frequency and the field level where a fault occurs. These differences can be explained by the fact that the operator did not detect the errors. The measurement uncertainties were 2.38 dB for this test. The maximum difference between the two methodologies is 2.3 dB. Therefore, the OVCV methodology can be validated.

With the results obtained on-vehicle and the results obtained on-table, we demonstrate that the validation of a complete EE architecture can be performed by the succession of simple component validation.

The traditional methodology has limitations that have been identified at the beginning of this paper. With the new OVCV methodology, we can validate EE architectures and automotive systems independently of their level of complexity. We can directly detect when two or several components have faults at the same frequency band but at different susceptibility levels, with high accuracy. With the traditional methodology, we cannot reach this level of accuracy and cannot directly detect this case (as described in Section 2). With the traditional methodology, we detect malfunctions of the component at the minimum susceptibility level. In addition, synchronization in the stimulation of several sensors is not necessary with the OVCV methodology. This allows us to avoid all the limitations of the traditional methodology and validate all the systems of an EE architecture with a succession of component validation tests with the OVCV methodology. Thus, the OVCV methodology does not require a metasystem for stimulation, which is highly costly. In addition, by using testing software, the EE architecture will be validated in fewer steps:

- Step 1: all central ECUs of the vehicle;
- Step 2: all sensors;
- Step 3: all actuators.

## 6. Conclusions

This study introduces a new approach for validating the radiated immunity of autonomous and advanced ADAS functions. All immunity tests were conducted in a semi-anechoic chamber. The proposed methodology relies on a step-by-step validation of individual components, enabling the validation of a complete architecture, regardless of its complexity. This is possible because synchronization of multiple sensors’ stimulations is no longer required, thanks to the use of test software. Dedicated test software would only need to be developed for each component. The methodology presented can be applied to validate any automotive electrical and/or electronic system.

In this paper, we presented the results of applying both the On-Vehicle Component Validation (OVCV) methodology and the traditional methodology to the same EE architecture. The results from both methodologies were compared, and we demonstrated that both approaches yield the same results, considering measurement uncertainties. However, the traditional methodology poses challenges for validating future autonomous functions and complex systems. In contrast, the OVCV method simplifies the validation of such complex systems. With the proposed methodology, it is not necessary to stimulate multiple sensors in a realistic or synchronous manner. The test software enables easy stimulation of several sensors simultaneously. Furthermore, the OVCV methodology allows for precise detection of faulty components without extensive investigation; it can identify susceptible components, even in cases where multiple faulty components exist in the same frequency band but for different field levels.

The limitations of the traditional methodology are overcome with the OVCV approach. Synchronizing sensor stimulation or achieving realistic sensor simulations are no longer required. Developing this new methodology will require cooperation between suppliers and automotive manufacturers. Additionally, we studied the correlation between on-table tests and on-vehicle tests in this paper. Thus, this new methodology does not require a stimulation metasystem or a significant amount of time to validate an entire complex EE architecture.

## Figures and Tables

**Table 1:** Example of six systems sharing six components embedded in a vehicle EE architecture.

	ECU1	ECU2	ECU3	ECU4	ECU5	ECU6
System 1	**x**	**x**		**x**		
System 2	**x**			**x**		
System 3	**x**				**x**	
System 4	**x**			**x**	**x**	
System 5	**x**		**x**			
System 6	**x**	**x**	**x**	**x**	**x**	**x**

**Figure A2:**
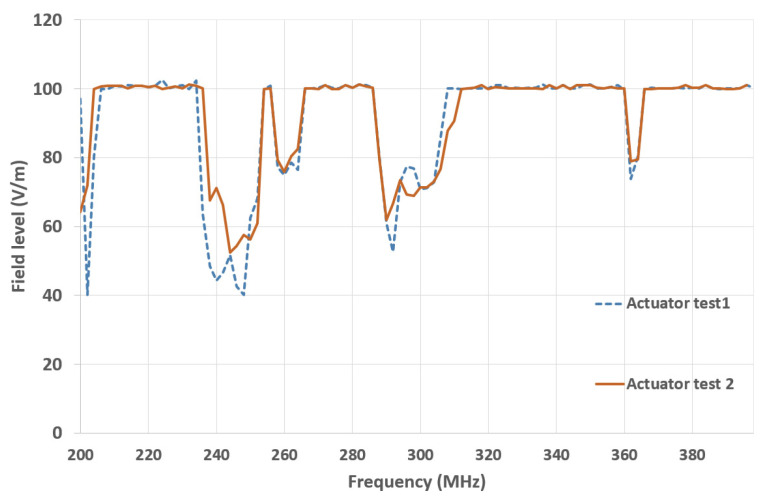
Susceptibility curves of the same component during two tests.

**Figure A3:**
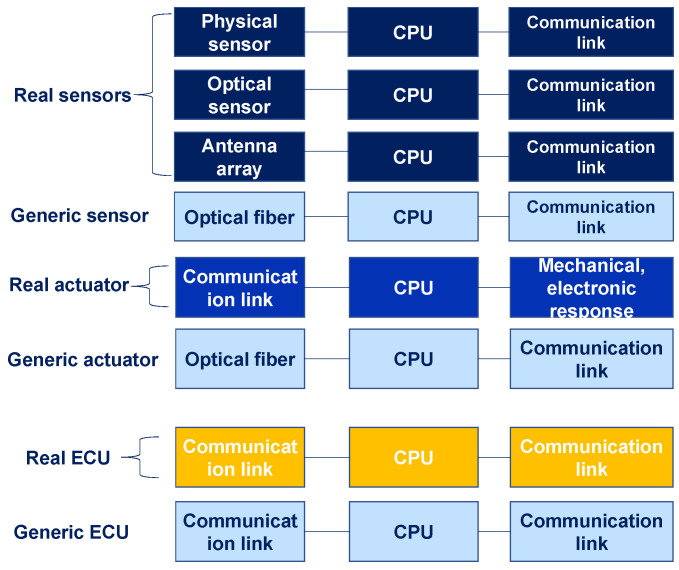
Analogy between real components and the generic components made.

## Data Availability

Data are contained within the article.

## Appendix A

To disturb several components, we decided to develop disturbance loops that are placed at the development card supply level. In Figure A2, the susceptibility curves of the ECU obtained with OVCV validation are presented. We can see through the susceptibility curve of the ECU without a disturbance loop that the generic component has no malfunctions in the studied frequency band. The susceptibility curve of the ECU with the disturbance loop demonstrates that we can master the susceptibility of the components depending on the loop we use, given the three different loops made the three generic components susceptible at different frequency bands. If we compare the susceptibility of ECU with the loops (Figure A1) and the susceptibility curves of the sensor and the actuator described in Figure 21, we notice that the three loops control the error level and frequency of each component. In a real case, these loops represent differences in susceptibility between components with different technologies. The validation of the sensor and the actuator without a loop was performed and the results are identical to the ECU without a loop: without a loop, we detect no malfunctions in the chosen components in the studied frequency band.

## Appendix B

In Figure A2, the susceptibility curves obtained during two tests of the same generic component with the same disturbance loop are illustrated. We validated the actuator performing the OVCV methodology during two tests on the same day, with less than five minutes between the two tests, with no change in the hardware and no access to the semi-anechoic chamber.

It can be seen in Figure A2 that we do not obtain the exact same results with the two tests.

These small differences are due to measurement uncertainties (2.38 dB) and can be explained by the fact that, during the threshold field level investigation, the field radiated is not exactly the same between the two tests.

## Appendix C

Figure A3 describes the analogy between real electronic components and the generic components made. The real sensors are composed of a physical sensor (e.g., antenna array for a radar), a CPU that processes data provided by the physical sensor, and a communication link. The generic components have an optical fiber that is equivalent to the physical sensor, a CPU, and a communication link which is equivalent to the real sensor. The generic actuators have a communication link, a CPU, and an optical fiber link equivalent to the mechanical or electronic part (the response) of a real actuator. The real ECU and the generic ECU have both a CPU and input and output communication links.
